# Diet and Skin Barrier: The Role of Dietary Interventions on Skin Barrier Function

**DOI:** 10.5826/dpc.1101a132

**Published:** 2021-01-29

**Authors:** Milbrey A. Parke, Ariadna Perez-Sanchez, Dina H. Zamil, Rajani Katta

**Affiliations:** 1 Baylor College of Medicine, Houston, TX, USA; 2 Internal Medicine, University of Texas Health Science Center at San Antonio, TX, USA; 3 Department of Dermatology, McGovern Medical School at UTHealth, Houston TX, USA

**Keywords:** skin barrier, supplements, atopic dermatitis, diet

## Abstract

Multiple research studies have examined the role of specific dietary interventions and their effects on skin barrier function. The skin barrier is one of the body’s first lines of protection against environmental insults, and disruption of this natural line of defense can result in xerosis, irritation, chronic dermatitis, and other cutaneous effects. Multiple laboratory, animal, and human studies have demonstrated that certain dietary interventions have the potential to impact skin barrier function. Measurements of skin barrier function include stratum corneum hydration and transepidermal water loss. In this review, we examine this research and provide an overview of the effects of prebiotics, probiotics, fatty acids, and emerging research on other substances.

## Introduction

The skin barrier is one of the body’s first lines of protection against environmental insults, including allergens, chemical irritants, microbial insults, and UV radiation. Disruption of this natural line of defense can result in xerosis, irritation, chronic dermatitis, and other cutaneous effects [1]. Given the skin barrier’s major role in providing protection from multiple threats, there has been much interest in strategies to improve and “strengthen” the skin barrier. A number of research studies have now examined the role of specific dietary interventions in enhancing skin barrier function. We review this research and provide an overview of the effects of prebiotics, probiotics, fatty acids, and emerging research on other substances.

## The Skin Barrier

### Background

While the skin barrier has traditionally been described in anatomic terms, it can also be described in terms of its components. Anatomically, the skin barrier can be divided into 2 layers, the dermis and epidermis. Functionally, Strugar et al. have described 4 main components: the physical layer, the microbiome, the immune layer, and the chemical layer. These components are all highly dependent on each other, and proper functioning of each component of the skin barrier is crucial for its protective role [2]. Disruption may lead to or exacerbate such conditions as irritant dermatitis, atopic dermatitis (AD), acne, and others [3,4].

The physical layer of the skin is composed of the stratum corneum, which is the outermost layer of the skin. Its main purposes include protection against water loss and against the penetration of irritants [5]. This layer can be further subdivided into cells (corneocytes) and a lipid matrix. The corneocytes secrete both the lipid precursors and the enzymes to modify these precursors into ceramides, essential and nonessential fatty acids, and cholesterol [6]. These lipids fill the extracellular space by forming lamellar bilayers, thus giving the skin its water-impermeable quality [6,7]. For the physical component of the barrier to function properly, it is believed that these lipids should be in a proportion of 50% ceramides, 25% cholesterol, and 10%–20% free fatty acids [8]. In addition, the omega-3/omega-6 fatty acid ratio is important for barrier function, with a higher ratio of omega-3 being more favorable [9]. It is also believed that a decrease in stratum corneum thickness and differentiation is part of the pathogenesis of sensitive skin [10].

The chemical layer, distributed throughout the physical layer, contains natural moisturizing factors, defense chemicals, and components that contribute to the acidic pH of the skin. The pH of healthy skin is between 4–6, and is important for the function of the many microbes that live on the skin [2]. At an alkaline pH, which may result from the use of some moisturizers, the microbes and enzymes of the skin do not function properly. An alkaline pH also activates Th2-producing endogenous serine proteases, which further disrupt the barrier [6]. Crucial to all of this is the protein filaggrin, which helps strengthen the corneocyte cell envelope, form lamellar bodies, and maintain the acidic pH [6].

The microbiome layer is composed of living organisms. Flora such as *Staphylococcus epidermidis* and *Corynebacterium* commonly predominate. However, disruption of the normally acidic chemical environment facilitates colonization by *Staphylococcus aureus* and *Staphylococcus pyogenes*, which may play a role in the pathogenesis of diseases such as atopic dermatitis [2,7].

The cells that make up the immune “layer” are always alert for danger signals, such as breaks in the skin barrier or signals sent by bacteria. If a threat is sensed, they can activate to repel microbes [2]. Skin barrier damage also triggers cytokine secretion, which then acts to regenerate the skin barrier by facilitating the growth and differentiation of keratinocytes [11,12].

## Measurement of the Skin Barrier

Many methods of measuring skin barrier function exist. Despite the many methods available, the optimal method of measurement is not well characterized, and conflicting results have been noted when evaluating the utility and accuracy of some methods. These uncertainties must be considered when evaluating the results of any dietary interventions on skin barrier function.

The most commonly used methods measure the physical layer of the skin barrier. The parameters targeted most often are stratum corneum hydration (SCH) and transepidermal water loss (TEWL). SCH measures the static water-holding capacity of the skin, while TEWL measures dynamic water losses [13]. Research indicates that both of these measures may be necessary to fully characterize the functioning of the skin barrier, and they are often used together. SCH is the amount of water contained in the stratum corneum. A few different methods are used to measure this parameter. These include electrical measurements such as capacitance, electrical impedance, and conductance [14]. Capacitance in particular measures surface hydration, but does not give information about the actual water content or thickness of the skin [5]. A newer method is Raman spectroscopy paired with multiphoton microscopy, which can measure full-thickness hydration of the stratum corneum [5,15]. Studies on evaluation of SCH reveal limitations of these measurement methods. Another measure of skin barrier function is that of transepidermal water loss (TEWL), which measures the insensible water loss through the skin and provides information on the lipid properties of the skin [14]. This method has been validated for in vivo study of skin permeability [16] although the utility of this method for in vitro testing is questionable.

## Synbiotics: Prebiotics with Probiotics

Synbiotics are a combination of probiotics and prebiotics, and their benefits are theorized to be due to effects on the gut microbiome. Among other effects, beneficial gut microbes produce short chain fatty acids, which help improve epithelial barrier function and modulate the inflammatory response [17,18]. Phenols, in contrast, are considered markers of a disturbed gut microbiome and have been demonstrated to disrupt keratinocyte differentiation in mice [19].

Probiotics are live microorganisms that are thought to have health benefits [20]. *Lactobacillus*, *Bifidobacterium*, and *Saccaromyces* species are among the most common microbes found in probiotics, and many but not all are found in human flora [21,22]. Prebiotics such as galactooligosaccharides (GOS) are substances which, when ingested, preferentially improve the growth of beneficial gut microbes.

Clinical studies of synbiotics in the treatment of AD have been promising, but little information is available on their skin barrier effects. In a meta-analysis of 6 studies, synbiotics with mixed bacterial strains were helpful for the treatment of AD in adults and children over the age of 1 year [23]. In a separate study, skin barrier function was measured. In this randomized controlled trial (RCT), consumption of the prebiotic GOS along with a probiotic milk for 4 weeks led to improved skin hydration. Serum phenol levels were also restored [24].

### Probiotics

The use of probiotics in treating inflammatory and infectious diseases of various organ systems has been of increasing interest, including in dermatology [25]. A potential role in the treatment of acne [26] and AD has been explored [27,28]. Their role in treatment may be in part due to their effect on the skin barrier.

The effect of probiotics on the skin barrier may be linked to their effects on immune regulation and the expression of skin barrier proteins. Studies in mice and skin models have shown decreases in pro-inflammatory cytokines, as well as improvement in skin hydration, TEWL, and the production of skin barrier proteins. In an ex vivo skin model, the topical application of both the lysate and whole preparations of a *Lactobacillus* species (*L. reuteri* DSM 17937) decreased production of IL-1 and IL-8 [25]. This also upregulated the gene for aquaporin 3, an important skin barrier protein that transports water and glycerol [25].

In a human epidermis model, the topically applied lysate of the probiotic *Lactobacillus rhamnosus* improved the expression of loricrin and filaggrin, both integral proteins of the skin barrier. This same strain decreased the destruction of desmosomes after treatment with the irritant sodium lauryl sulfate [29].

Despite these promising results, direct research on the effect of probiotics alone on the skin barrier function in humans is relatively lacking. In one RCT, skin hydration was increased and TEWL was decreased after 12 weeks of oral *Lactobacillus plantarum* HY7714[30]. Another RCT evaluated the effects of 2 months of oral *Lactobacillus paracasei* NCC2461, and found improved skin barrier function recovery, as indicated by TEWL after tape stripping [31]. Another RCT compared the effects of *Lactococcus lacti* H61 consumption in milk compared to a conventional probiotic yogurt for 4 weeks. Both groups had an increase in skin hydration, and the probiotic milk group also had a significant increase in sebum content [32].

### Prebiotics

Limited studies are available specifically examining the role of prebiotics. In one RCT, healthy adults consuming GOS for 12 weeks had greater improvement in TEWL and skin hydration compared to the placebo group [33]. In mice with allergen-induced dermatitis, treatment with GOS improved both clinical severity and TEWL [34].

Overall, more human studies are needed to establish a clear association between probiotic and prebiotic consumption and skin barrier function. Human studies to date are small. However, given promising results, further research is warranted.

## Omega-3 Fatty Acids

Omega-3 fatty acids include alpha-linolenic acid (ALA), eicosapentaenoic acid (EPA), and docosahexaenoic acid (DHA). Derivatives of omega-3 fatty acids are thought to influence the skin barrier by acting as transcription factors and decreasing inflammation as immune modulators [35].

Studies in skin models and cultured human keratinocytes suggest that DHA can increase filaggrin expression, attenuate inflammation, and improve epidermal keratinocyte differentiation [36–38]. An animal study demonstrated decreased TEWL, increased skin hydration, and decreased skin barrier alteration by acetone [39]. EPA and the ester of EPA have been shown in animal studies to influence ceramides in skin [40,41]. However, studies have utilized different methodologies and have shown varying improvement [36–38].

Several small human trials have examined the effects of oils rich in fatty acids. Flaxseed oil, high in ALA, was shown to improve TEWL, skin hydration, skin scaling, and roughness in female study subjects after 12 weeks of daily consumption [42]. In another study, improvements in TEWL, skin hydration, and skin sensitivity were noted [9]. In both studies, the proportion of plasma ALA in those consuming flaxseed oil increased.

Another RCT evaluated hempseed oil ingestion in those with AD. Hempseed oil contains both omega-6 and omega-3 fatty acids, in a ratio of about 2:1. Subjects reported improved skin dryness and less use of dermal medications with the hempseed oil. Although there was a trend to improved TEWL, this was not significant [43].

Limited human trials have studied the effects of fish oils specifically on TEWL. One RCT found that use of DHA for 8 weeks led to significant improvement in clinical findings of AD and an increase in plasma levels, but did not evaluate TEWL [44]. Another RCT found that a 4-month supplementation with fish oils led to an overall 30% improvement in clinical AD scores, although this was similar to a comparison group given corn oil. Of note, both groups had a baseline of significantly lower levels of serum fatty acids compared to patients with psoriasis [45].

While these studies are small, they suggest that consumption of oils rich in omega-3 fatty acids have the potential to improve skin barrier function, possibly in those with lower serum levels at baseline. Larger studies in humans are needed to assess the magnitude of effect of these treatments.

## Evening Primrose and Borage Oils

Evening primrose oil (EPO) and borage oil (BO) have been studied for the treatment of AD, with overall disappointing results [46]. However, while many AD studies have focused on measures of clinical disease severity, few have investigated their effects on skin barrier function directly. Of these, some have reported promising results.

Gamma-linolenic acid (GLA) is an omega-6 fatty acid that has exhibited beneficial properties. EPO contains 8%–10% GLA and BO contains up to 25% GLA. [47,48]. One study described a negative correlation between TEWL and serum levels of GLA in children with AD. The study also noted a correlation between barrier dysfunction severity and deficiencies in essential omega-6 fatty acids such as GLA [49,50].

Human skin lacks the enzyme delta 6-desaturase, which converts linoleic acid (LA) to GLA [51–53]. As such, the skin depends on GLA created in the liver [53]. Oral supplementation of EPO and BO supplies GLA directly [47,48].

It is believed that the benefits of EPO and BO may be due to the influence of GLA and its metabolites on regulatory mechanisms of barrier function [53]. Metabolites of GLA exhibit anti-inflammatory properties and are linked to increased ceramide synthesis and improved skin barrier function [47,49,54,55]. They also inhibit the formation of leukotriene (LT)B_4_ [49,56–58].

Clinical studies of EPO and BO have varied widely, with differing populations and doses. However, multiple studies have demonstrated significant beneficial effects on TEWL [42,53,55,59]. Stratum corneum hydration, overall, has not shown significant increases [53,55,60].

Negative results have been reported as well, as 2 placebo-controlled trials found no differences in skin barrier function after GLA supplementation [49,60]. One of these, however, did find significant improvement in TEWL in a subgroup. These patients at baseline had low serum levels of a GLA metabolite, suggesting the importance of baseline status in determining who might benefit from supplementation [49].

Overall, while EPO and BO supplementation has not shown benefit for the treatment of AD, they have shown some promise in enhancing skin barrier function. Further research should examine baseline levels of GLA that may benefit from supplementation, as well as comparing dosing regimens.

## Emerging Interventions

### L-Histidine

Limited research has been performed on l-histidine supplementation, but results from 1 trial are promising and further research is warranted. L-histidine is an amino acid that is rapidly incorporated into filaggrin. The pathogenesis of AD has been linked to deficiencies in filaggrin, with loss of function mutations in filaggrin genes showing a strong association with AD [61]. In one small RCT with 24 adult AD patients, once-daily oral l-histidine for 4 weeks significantly increased both filaggrin formation and skin barrier function. AD severity as measured by SCORAD improved as well, with no improvement in the placebo group [62].

### Combination Products

While some combination products have shown a reduction in TEWL, the limited available research, small sample sizes, and use of multi-ingredient products limit our conclusions. This includes studies on a combination turmeric and herbal product [63], a combination borage oil, fermented milk, and vitamin product [54], and a proprietary fish oil blend with vitamin D, antioxidant mixture, and other oils [64].

## Conclusions

Several dietary interventions have been studied for their potential impact on the skin barrier, primarily in the form of dietary supplements. Laboratory and animal studies have described potential mechanisms of action. While some benefits have been seen in clinical studies, a number of questions remain. Clinical trials to date have utilized small sample sizes, and many have used varying dosing regimens. Some studies have evaluated multiple active ingredients, making it difficult to isolate the effects of a single ingredient. In addition, several interventions have been studied in disease states, and therefore may not be applicable to those without skin disease. Some studies have found that a baseline status of nutrients impacts clinical outcomes and is an important variable that should be studied further in future clinical trials. It is notable that few studies have investigated the role of dietary patterns on skin barrier function. This is an important area for future study. Research studies have indicated, for example, that changes in diet can impact the gut microbiome within 1 day [65], with potential implications for the skin barrier. Current research has been focused on interventions with dietary supplements and a few foods, but further research on the effects of foods and dietary patterns on the skin barrier is warranted. Although questions remain about dosing, duration, and which populations may benefit, results to date for some interventions have been promising and further research is appropriate.
